# MicroRNA124 and microRNA21-5p regulate migration, proliferation and differentiation of rat bone marrow mesenchymal stem cells

**DOI:** 10.1042/BSR20193531

**Published:** 2020-10-23

**Authors:** Yan Liu, Xiaofu Zhang, Chao Gao, Hang Zhang, Hongtao Zhang, Jing Qu

**Affiliations:** 1 Department of Orthopedics, the First Affiliated Hospital of Soochow University, Soochow University, Suzhou, Jiangsu, China; 2 Department of Cell Biology, Jiangsu Key Laboratory of Stem Cell Research, Medical College of Soochow University, Ren Ai Road 199, Suzhou Industrial Park, Suzhou 215123, China

**Keywords:** Differentiation, Migration, MiRNA124, MiRNA21, MSCs, Proliferation

## Abstract

Mesenchymal stem cells (MSCs) are multipotent stromal cells that can be a useful source of cells for the treatment of many diseases, including neurologic diseases. The curative effect of MSCs relies mostly on cell’s capacity of migration, proliferation and differentiation. MicroRNAs (miRNAs) are small non-coding RNAs that play important roles on regulating various cell behaviors. Here, we report that miRNA-124 (miR124) and miRNA-21-5p (miR21-5p) display different regulatory roles on migration, proliferation and neuron differentiation of MSCs. MiR124 was shown greatly promoting MSCs migration and neuronal differentiation. MiR21-5p could significantly enhance the proliferation and neuronal differentiation ability of MSCs. MiR124 and miR21-5p synergistically promote differentiation of MSCs into neurons. Collectively, miR124 and miR21-5p can functionally regulate cell migration, proliferation and neuronal differentiation of MSCs. Therefore, miR124 and miR21-5p may be promising tools to improve transplantation efficiency for neural injury.

## Introduction

Mesenchymal stem cells (MSCs), characterized by multi-potent differentiation, are a stem cell type found in the bone marrow, adipose and other tissues. It becomes an attractive cell source for regenerative medicine applications due to their good proliferation and differentiation abilities [1]. MSCs can be cultured *in vitro* for many passages and are widely used in clinical applications [2], including treating liver disease [3] and neurologic diseases [4]. Nowadays, many people are suffering from neurologic diseases such as Spinal cord injury (SCI), neurodegenerative diseases and central nervous system (CNS) tumors. Among these diseases, SCI is a devastating disease, because mammals are unable to regenerate their spinal cords after injury. Patients and their families are often deprived of the quality of their lives forever [5]. So far, there is no effective cure for SCI and the promising methods for the treatment of SCI including conventional treatment, stem cell transplantation and gene therapy [6].

In recent years, more and more attention has been paid to the treatment of SCI by stem cells. These cells can not only release neurotrophic factors, but also regenerate injured nerve tissue through differentiation into neural cells [7]. Among these cells, MSCs have gained growing interest in cell therapy because it has multiple differentiation and proliferation capacity, present low immunogenicity, and are easy to harvest, culture and amplify too. It has become a useful stem cell source for the treatment of SCI [7–10]. Furthermore, MSCs show a high expression of growth factors, such as hepatocyte growth factor (HGF), brain-derived neurotrophic factor (BDNF), neural growth factor (NGF), vascular endothelial growth factor (VEGF), insulin-like growth factor 1 (IGF-1), glia cell-line derived neurotrophic factor (GDNF), cytokines, and extracellular matrix molecules, all these play important roles in nourishing and protecting neurons [5,9,11]. Also, many studies suggest that MSCs can differentiate into neuronal-like morphology exclusively [12], which overcomes the risks of harvesting neural stem cells from the brain, and provide a renewable population of MSCs. In recent years many experimental studies have proved that MSCs can reverse functional deficits when they were transplanted locally, intravenously, or intra-arterially [13]. Moreover, MSCs are reported to differentiate into cells that were immunopositive for microtubule-associated protein 2 (MAP-2), 2′,3′-cyclic nucleotide-3′-phosphodiesterase (CNPase) and glial fibrillary acidic protein (GFAP) after being administered into rat [14]. Although these preliminary findings may seem promising, further research is needed. As it is reported that after intravenous transplantation, the labeled MSCs were seen colonized more in the spleen, liver and kidneys, only a few MSCs reached the SCI area [15]. It is important to make sure that the cells migrate into the injured area, stay alive for a long time and differentiate into neurons at the injured area [9].

In addition to cell therapy, the regulation of miRNAs in gene therapy has attracted more and more attention in recent years [15], and it may provide better therapeutic strategies for SCI treatment. MiRNAs are small non-protein-coding RNAs composed of 20–23 nucleotides and have been identified to be important in the regulation of cell immigration, proliferation, apoptosis, differentiation, metabolism and tumorigenic transformation [16–20]. MiR124 is expressed abundantly in brains of mature mammals and is one of the earliest highly conserved miRNAs ever found. It plays an important role in neurogenesis [4]. MiR124 can be transferred from neurons to astrocytes via exosomes *in vivo* and that acts non-cell autonomously to regulate astroglial glutamate uptake function and maintain axon growth [21]. It was reported that the cell behavior of MSCs is closely related to the expression of miR124 [22,23], and miR124 was shown to play an important regulatory roles in functional recovery after SCI [24]. MiR124 treatment can significantly increase the intracellular expression levels of the neuronal early markers: β3-Tubulin (TUJ-1) and MAP-2 [25,26]. It has also been reported that MSCs can functionally deliver exogenous miR124 to neural cells and that increases the neuronal differentiation of neural progenitor cells (NPCs) and the expression of glutamate transporters in NPCs and astrocytes [27]. Therefore, further understanding of the mechanism of miR124 in regulating migration and proliferation will help to improve the application of MSCs as therapeutic vehicles.

MicroRNA-21 (miRNA21) was reported to play functional roles to regulate anti-apoptosis, proliferation and migration behaviors of many kinds of cells [28,29]. After traumatic brain injury (TBI), the expression level of miRNA21-5p in the brain was increased, which inhibited nerve cell apoptosis and promoted angiogenesis in the brain, and protected the blood–brain barrier (BBB) injury, thereafter improved the function of the nervous system of TBI in rats [30]. The expression of miR21-5p was significantly increased after peripheral nerve and dorsal root ganglion (DRG) neurons injury. MiR21-5p was further confirmed to enhance neurite growth and play neuroprotection functions [31–34]. It was also shown that miR21-5p could regulate the cell apoptosis process after SCI by regulating its target gene phosphatase and tensin homolog (PTEN) [35]. In addition, there are studies indicated that miR21-5p may have the ability to protect MSCs from injury and may be beneficial to stem cell therapy after SCI [28]. In the present study, we made efforts to explore the regulatory roles of miR124 and miR21-5p on MSCs migration, proliferation and neuronal differentiation and hope to provide fundamental data for future clinical application of MSCs to promote structural and functional recovery efficiently after SCI**.**

## Materials and methods

### Isolation and culture of MSCs

All experimental procedures were approved by the Institutional Animal Care and Use Committee of Soochow University. Sprague–Dawley rats (SD rats) were used in all experiments and were purchased from Zhao Yan company (Suzhou, co. LTD, China). All animal experiments are carried out in the Animal Experiment Center of Soochow University. Cells from the bone marrows of 4-week-old SD rats (weight, 80–100 g, *n*=9) were isolated and cultured as reported previously [36]. Briefly, rats were killed by cervical dislocation after anesthetized with chloral hydrate (400 mg/kg, i.p.). Then, the lower limbs were shaved and soaked in 75% ethanol for 10 min. The tibias and femurs from both legs of each rat were dissected and the two ends of each bone were cut under aseptic conditions. The marrow was flushed out with a medium consisting of Dulbecco’s modified Eagle’s medium/Low glucose (L-DMEM, Hyclone), penicillin (100 U/ml) and streptomycin (100 U/ml; Sigma, St. Louis, U.S.A.). The bone marrow cells flushed with the medium were gently drawn up and down with a 5-cc syringe and 21-gauge needle, and then a single cell suspension was obtained. After the bone marrow was centrifuged at 500 × *g* for 10 min at 4°C, cells were adjusted to 1 × 10^6^ cells/ml and seeded in 100 mm cell flasks containing L-DMEM supplemented with 10% fetal bovine serum (FBS) (Hyclone, Logan, U.S.A.), 1% penicillin and streptomycin and incubated at 37°C with 5% CO_2_ in a humidified incubator. Half of the culture medium was refreshed 48 h after the seeding. Then refresh the culture medium every 48 h. About 10 days later, when cells were confluent, the cells were passaged with trypsin (Hyclone, Logan, U.S.A.). MSCs at passage 3 through 10 were used for the experiments.

### MSCs treatment

MSCs were resuspended and seeded at 5 × 10^4^cells/ml into six-well culture plates and incubated at 37°C before transfection. MSCs were divided into four groups. according to instructions provided by the manufacturer, when the cell density get to 80%, 50 nM synthetic miR124 mimics (RiboBio, Guangzhou, China) were transfected by Lipofectamine 2000 (Invitrogen, Carlsbad, CA) into MSCs to overexpress miR124 in MSCs and this group is defined as miR124-MSCs group. In the same way, miR21-5p mimics (RiboBio, Guangzhou, China) and miRNA mimics negative control (RiboBio, Guangzhou, China) were respectively transfected into MSCs and they were respectively defined as miR21-5p-MSCs group and mimics control-MSCs group (MC-MSCs). The miR124 mimics and miR21-5p mimics were co-transfected into MSCs that was named as the miR124+21-5p-MSCs group. In addition, MSCs treated with L-DMEM only was also used as a control group that was defined as normal control-MSCs group (NC-MSCs). After incubation for 48 h at 37°C with 5% CO_2_ in a humidified incubator, the cells were collected for the further experiments. All the miRNA mimics used in the study are listed in Table 1 and the experiments were performed in triplicates and repeated in three independent experiments.

**Table 1:** All the primers used in this experiment

Items	Sequences
miR124 mimics	(5′-3′): UAAGGCACGCGGUGAAUGCC
miR21-5p mimics	(5′-3′): UAGCUUAUCAGACUGAUGUUGA
miRNA mimic negative control	(5′-3′): UUUGUACUACACAAAAGUACUG
miR124 qRT-PCR primer	(5′-3′): UAAGGCACGCGGUGAAUGCC
MiR21-5p qRT-PCR primer	(5′-3′): UAGCUUAUCAGACUGAUGUUGA
MiRNA mimie negative control qRT-PCR primer	(5′-3′): UUUGUACUACACAAAAGUACUG

### Real time polymerase qRT-PCR reaction

To examine the expression of miR124 and miR21-5p in the MSCs, we used quantitative real-time polymerase chain reaction (qRT-PCR) to measure their miRNA levels. Briefly, total RNAs were isolated with Trizol reagent (Invitrogen) and reverse transcribed to cDNA following the manufacturer’s protocol (Invitrogen). QRT-PCR was carried out with Bulge-Loop™ miRNA qRT-PCR Starter Kit (R11067.2, RiboBio, Guangzhou, China) with the following parameters: pre-denaturation at 95°C for 10 min, followed by 40 cycles of denaturation at 95°C for 2 s, annealing at 60°C for 20 s and extending at 70°C for 10 s. QRT-PCR reactions were performed in the PTC-220 Real-Time PCR Machine (Bio-rad). The primers used in the study are listed in Table 1 and the experiments were performed in triplicate. The levels of miR124 and miR21-5p were normalized against U6 snRNA (MQP-0202, RiboBio, Guangzhou, China) and we calculated the result with the 2^−ΔΔCt^ method.

### Wound healing assay

Each group of MSCs was resuspend and seeded at a density of 5 × 10^5^ cells/well in the six-well plates and monolayer cells concentrated after 24 h culture using complete medium. Then, the monolayer cells were wounded with a sterile 200-μl pipette tip. The cell debris was washed away with PBS and the medium in the dishes were changed to L-DMEM without FBS. The dishes were incubated in incubator at 37°C with 5% CO_2_ atmosphere [37]. The cell migration from the edge of the scratch to the middle was monitored regularly at 0, 6, 12, 24 h after wounded and the digital images of at least nine random areas in the scratch area were photographed using an inverted phase contrast optical instrument (ZEISS, Germany) [38]. The wound area at each time point was measured with ImageJ software. Three replicates were undertaken for each assay. The data were expressed as mean ± SEM deviation.

### The transwell migration assay

Migration ability of MSCs was assessed using the Corning Costar 24-well Transwell chamber system Assay Kit (8 μm size), which calculated the number of cells that passed through a polycarbonate membrane. About 200 μl serum-free L-DMEM medium containing 1 × 10^5^ cells were added to the upper chamber. A volume of 500 μl of 10% FBS-containing L-DMEM medium was then added to the lower chamber as a chemoattractant. After incubated at 37°C for 24 h, the non-migrating cells on the upper surface were carefully scraped off with cotton swabs. Cells that migrated to the bottom of the membrane were fixed with 4% paraformaldehyde, soaked with PBS, and stained with 1% Crystal Violet. Stained cells were visualized and counted under a microscope. To minimize the bias, three randomly selected fields were quantified, and the average number of cells was taken. Three experiments were repeated for each group.

### Cell proliferation assay

The proliferation assay of MSCs was determined by using the Cell-Light™ EdU Apollo®488 In Vitro Imaging Kit (RiboBio, Guangzhou, China) according to the manufacturer’s protocol. Briefly, MSCs were resuspend and seeded at a density of 1 × 10^4^ cells/well in the 96-well plates. When the cell density get to 80%, MSCs were cultured with 10 μM EdU for 3h at 37°C with 5% CO_2_ in a humidified incubator before fixation, permeabilization, and EdU staining. EdU (5-ethynyl-20-deoxyuridine) is a nucleoside analog of thymidine that is incorporated into DNA during active DNA synthesis only by proliferating cells. After incorporation, a fluorescent molecule was added that reacted specifically with EdU, making possible fluorescent visualization of proliferating cells. Cell nuclei were stained with Hoechst 33342 (Invitrogen) at a concentration of 1 μg/ml for 30 min. After EdU and Hoechst 33342 staining, the digital images of at least nine random areas in the plate were photographed using an inverted phase contrast optical instrument (ZEISS, Germany). Then the number of the EdU staining positive cells and the total cells indicated with Hoechst 33342 staining positive were each counted. Three replicates were undertaken for each assay. The data were expressed as mean ± SEM deviation.

### Neuronal differentiation of MSCs

MSCs were induced to neuronal differentiation with the method modified on Woodbury et al. [12]. First, MSCs were resuspend and seeded at a density of 1 × 10^5^ cells/well in the 24-well plates and that were cultured in L-DMEM/10% FBS for 24–48 h. Then, the culture media were replaced with pre-induction culture media consisting of L-DMEM/20% FBS/1 mM β-mercaptoethanol (BME) and it would maintain for 24 h. After that, the pre-induction culture media were removed, and the cells were washed with PBS three times. Then the induction media, which was composed of L-DMEM/2 mM BME/2% dimethylsulfoxide (DMSO)/200 μM butylated hydroxyanisole (BHA), was added to culture cells for another 5 h. At last, the cells were maintained in H-DMEM/1%N2 for 24 h before they were fixed for immunocytochemistry.

### Immunocytochemistry

Cultured MSCs were fixed on culture dishes by treating with 4% buffered paraformaldehyde for 10 min. The samples were blocked with 5% Bull Serum Albumin (BSA, Solarbio, Beijing, China) in PBS for 60 min, and incubated with primary antibody (TUJ-1, β3-Tubulin (D71G9) XP® Rabbit mAb #5568, CST, diluted in 1:500; Neuronal nuclear antigen (NeuN) (D4G4O) XP® Rabbit mAb #24307, CST, diluted in 1:500) overnight at 4°C. The cells were incubated with secondary antibody (Goat anti-Rabbit IgG (H+L) Highly Cross-Adsorbed Secondary Antibody, Alexa Fluor 488, Invitrogen **#** A-11034 diluted in 1:1000) for 60 min at room temperature then incubated with Hoechst 33342 (C1025, Beyotime, China) for 15 min. Between every step, the cells were carefully washed with PBS three times. For quantification, a camera (ZEISS, Germany) was used to capture at least nine non-overlapping images (×200) of each marker. The cells with NEUN or TUJ-1 staining positive were counted as percentage of total cell counts. Three replicates were undertaken for each assay. The data were expressed as mean ± SEM deviation.

### Statistical analysis

All measurement data are expressed as the mean ± SEM deviation. SPSS 17.0 software (SPSS, Chicago, IL, U.S.A.) was used to analyze the date. Student’s *t*-test and one-way analysis of variance was used for comparison of the mean among groups. A level of *P* < 0.05 was considered statistically significant.

## Results

### MiR124 and miR21-5p are significantly up-regulated in MSCs

**Figure 1:**
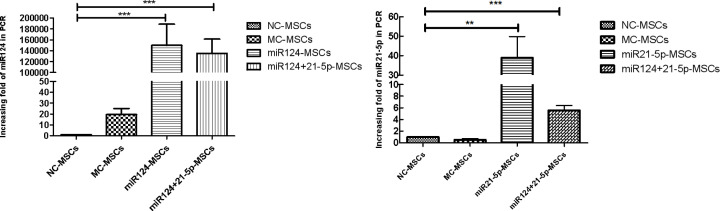
The expression of miR124 and miR21-5p were both significantly increased in MSCs MiR124 mimics, miR21-5p mimics and control miRNA mimics were transfected into cells. Total RNAs were isolated and qRT-PCR was carried out. The results were calculated with the 2^−ΔΔCt^ method. After the transfection treatment, the expression of miR124 (***, *P*<0.0001) or miR21-5p (**, *P*=0.0026, <0.01) was significantly increased compared with the NC-MSCs group. For the miR124+21-5p-MSCs group, the expression of miR124 (***, *P*<0.0001) and miR21-5p (***, *P*=0.0005, <0.001) were both significantly increased compared with the NC-MSCs groups (sample size: 18).

In order to investigate the role of miR124 and miR21-5p in the cell behavior of MSCs, we determined the differential expression of miR124 and miR21-5p as previously mentioned. We performed qRT-PCR to quantitate the expression levels of miR124 and miR21-5p 48h after MSCs were transfected. The results showed that it is about 150,000-fold higher for the miR124 (**, *P*=0.001, <0.01) and 40-fold higher for the miR21-5p (*, *P*=0.0129, <0.05), comparing to the NC-MSCs group, indicating that miR124-mimics and miR21-5p-mimics significantly up- regulated miR124 or miR21-5p expression in MSCs (Figure 1).

### Overexpression of miR124 promotes migration of MSCs

**Figure 2:**
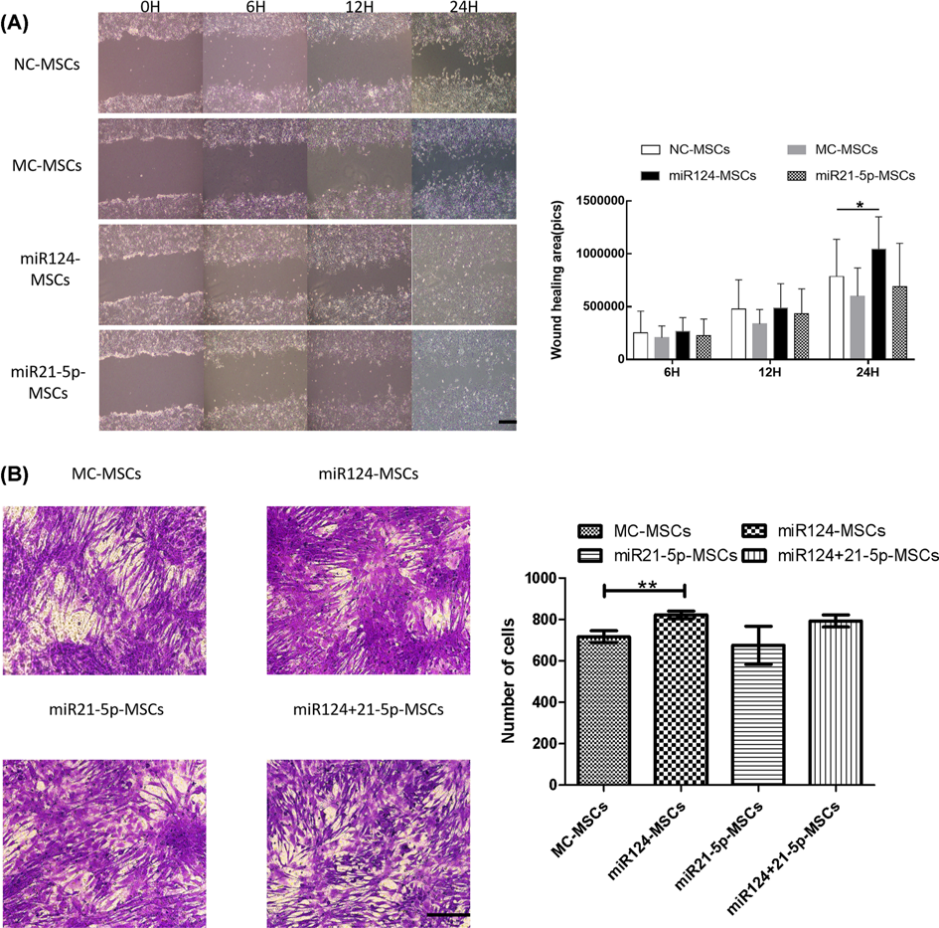
Overexpression of miR124 promotes migration of MSCs (**A**) The monolayer cells were wounded with a sterile 200-μl pipette tip. Images of the scratched area were taken 0, 6, 12, 24 h after treatment. The photos were measured with ImageJ software. NC-MSCs group cells started to migrate into the denuded area at 6 ho after being scratched, and scratch closure was almost (∼50%) complete at 24 h. In contrast, miRNA124-MSCs cells migrated more quickly, as indicated by more cells in the denuded area at 24 h after scratching. In addition, the miR21-5p-MSCs group showed no effect on cell migration at any time point than the NC-MSCs group. Our data demonstrated that comparing to the NC-MSCs-24h group, the miR124-MSCs-24h group showed significantly much more wound healing area (*, *P*=0.0384, <0.05) (Sample size:12; scale bar = 200 μm). (**B**) Cells that passed through the polycarbonate membrane were stained with Crystal Violet, observed under an inverted phase contrast microscope. The histogram showed the number of cells that passed through Transwell migration chambers. In the miR124-MSCs group, there are much more MSCs passed through the chambers (**, *P*=0.0077, < 0.01). All of the data were presented as the mean ± SEM of three independent experiments (sample size:12; scale bar = 200 μm).

To determine the effects of miR124 and miR21-5p on cell migration in MSCs, we carried out cell wound healing experiments and the Transwell migration assay. In the wound healing experiment, cells in the NC-MSCs group began to migrate into the scratched area within 6 h after being wounded, and the denuded area was almost half closed at 24 h (Figure 2A). Compared with the NC-MSCs group, cells in the miR124-MSCs group migrated more quickly, as indicated by more cells in the denuded area at 24 h after scratching (Figure 2A). Moreover, cells in the miR21-5p-MSCs group showed no significant effect on cell migration at any time point comparing to the NC-MSCs group (Figure 2A). We calculated the wound healing area of each group at each time point with ImageJ software. The results indicated that miR124-MSCs-24h group showed significantly more wound healing area comparing to the NC-MSCs-24h group (*, *P*=0.0384, <0.05). Transwell migration assay was also used to assess the effect of miR124 and miR21-5p on MSCs migration. According to the results of the assay, the number of migrated cells in MC-MSCs group, miR124-MSCs group, miR21-5p-MSCs group and miR124+21-5p-MSCs group was 716.5 ± 28.79, 822.5 ± 18.26, 675.0 ± 92.08 and 793.3 ± 29.15, respectively. According to the statistical analysis, the number of migrated cells significantly increased in miR124-MSCs group compared with the control group (**, *P*=0.0077, <0.01), while there was no significant difference between other groups (Figure 2B).

### Overexpression of miR21-5p promotes proliferation of MSCs

**Figure 3:**
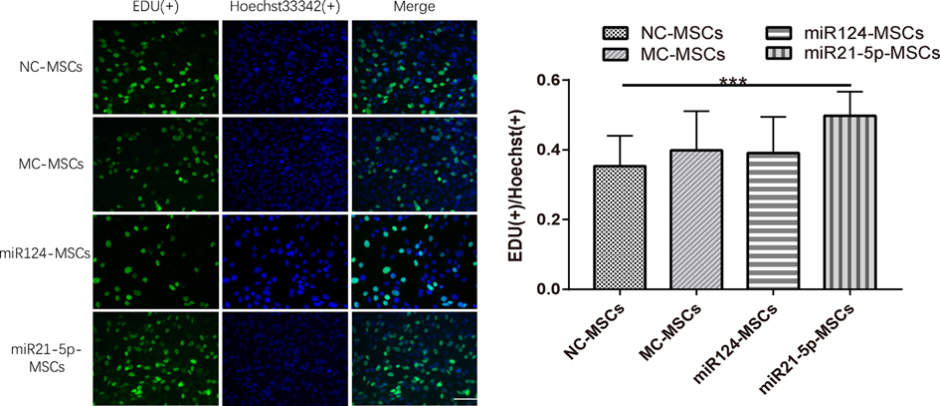
Overexpression of miR21-5p promotes proliferation of MSCs Cells were incubated with EDU before EDU staining. Cell nuclei were stained with Hoechst 33342. EDU is positive only in proliferating cells. Images of at least nine random fields of the plates were taken using inverted phase contrast optics. Then the number of the EDU staining cells and the total cells were each counted and the rate of EDU(+)/Hoechst(+) was measured. Overexpression of miR21-5p promotes MSCs proliferation while overexpression of miR124 have no effect on proliferation. The rate of the miR21-5p-MSCs group had a significant difference with the NC-MSCs group (***, *P*<0.0001) while others shown no differences (sample size:12; scale bar = 50 μm).

To investigate the effects of miR124 and miR21-5p on proliferation in MSCs, we took cell proliferation assay. As it is shown on Figure 1, miR124 mimics and miR21-5p mimics increased miR124 and miR21-5p expression levels in MSCs. Under these conditions, the quantitative analysis measured by EdU (+) / Hoechst 33342 (+) showed that overexpression of miR21-5p in MSCs promotes cell proliferation ability while overexpression of miR124 have no significant effect on cell proliferation ability (Figure 3). The rate of the miR21-5p-MSCs group had a significant difference with the NC-MSCs group (***, *P*<0.0001) while the miR124-MSCs group, the MC-MSCs group and the NC-MSCs group showed no significant difference between each other (Figure 3).

### Characterization of the neuronal differentiation of MSCs

**Figure 4:**
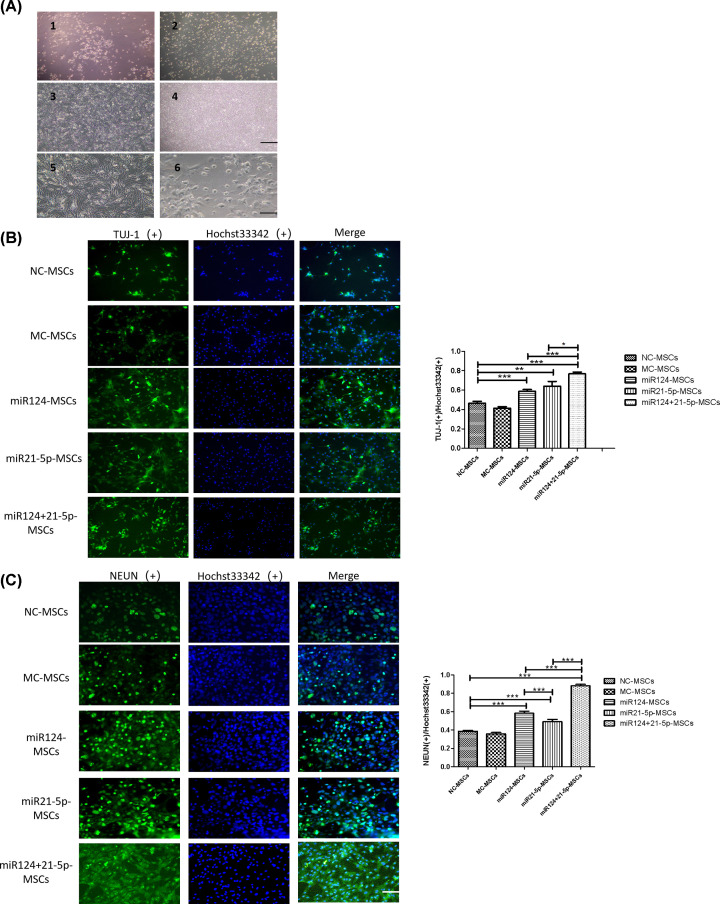
Overexpression of miR124 and miR21-5p significantly promotes neuronal differentiation of MSCs (A-1) Culturing the cells for 5 days, only a few cells were triangular-shaped. (A-2) Culturing the cells for 14 days, the cells converged into a colony. (A-3) After three passages, morphologically homogenous populations of fibroblast-like cells were observed. (A-4) After ten passages, the cells still grow with fibroblast like shape and gathering to form a vortex (the cells in A1-4 were from NC-MSCs group. scale bar = 200 μm). (A-5) Flat MSCs is identified prior to differentiation. (A-6) After differentiation, the MSCs were changing to neural-like cells which display condensed cell bodies and highly branched processes (The cells in E-F were from miR124-MSCs group) (scale bar = 100 μm). (**B** and **C**) MSCs were fixed on culture dishes. Then, the samples were blocked and stained with antibodies against NEUN and TUJ-1. Then the cell nucleus were stained with Hoechst 33342. Different groups showed different staining positive proportions. The proportion of TUJ-1 (+) in miRNA124+21-5p-MSCs group (76.8%±1.9%) is the highest, followed by miRNA21-5p-MSCs group (64.0%±4.9%) and miRNA124-MSCs group (58.9%±1.8%). MiRNA21-5p-MSCs group (**, *P*=0.0045, <0.01), miRNA124-MSCs group (***, *P*=0.0002, <0.001) and miRNA124+21-5p-MSCs group (***, *P*<0.0001) have statistical difference compared with NC-MSCs group in the proportion of TUJ-1 (+). The proportion of TUJ-1 (+) in the miRNA124+21-5p-MSCs group was higher than that in MiRNA21-5p-MSCs group (*, *P*=0.0178, <0.05) and miRNA124-MSCs group (***, *P*<0.0001). MiRNA124+21-5p-MSCs group (88.3% ± 1.5%) has the highest proportion of NEUN (+), followed by miRNA124-MSCs group (58.3% ± 2.3%) and miRNA21-5p-MSCs group (49.1% ± 2.6%). MiRNA124-MSCs group, miRNA21-5p-MSCs group and miRNA124+21-5p-MSCs group have statistical difference compared with NC-MSCs group in the proportion of NEUN (+) (***, *P*<0.0001). The proportion of NEUN (+) in the miRNA124+21-5p-MSCs group was higher than that in MiRNA21-5p-MSCs group (***, *P*<0.0001)and in miRNA124-MSCs group (***, *P*<0.0001) (sample size:30; scale bar = 50 μm).

Cells started to attach to the flasks 3 h after seeding. The number of the attached cells increased after changing the culture medium 48 h later. The culture medium was changed every 48 h. After 5 days of cell culture, most of the cells grew in a spindle-shaped spiral, and only a few cells showed a triangular shape. After three passages of amplification, morphologically homogenous populations of fibroblast-like cells were observed and this cell morphology would maintain for more than ten passages (Figure 4A1–4).

To determine the effects of miR124 and miR21-5p on neuronal differentiation in MSCs, we carried neuronal differentiation and immunocytochemistry experiments. MSCs were induced to differentiate into neurons with the protocol mentioned above, and different proportions of cells undergo neuronal differentiation in different groups. In this experiment, all TUJ-1 and NEUN positive cells generally accounted for 51.4% of the total cells. During the differentiation process, cytoplasm in the flat MSCs gradually retracted toward the nucleus, forming a contracted multipolar, cell body, leaving membranous, process-like extensions peripherally (Figure 4A5–6). After the induction of neuronal differentiation, the cells were stained with neuronal markers NEUN and TUJ-1, and the percentage of neurons in the cells after differentiation was determined. A lot of the treated MSCs exhibited neuronal morphology and the average positive expression ratio for NEUN and TUJ-1 of all the cells in our experiment are 48.3% ± 1.7% and 58.3% ± 2.2%.

### Overexpression of miR124 and miR21-5p significantly promotes neuronal differentiation of MSCs

With the foregoing induction protocol, a variable number of cells underwent neuronal differentiation in different groups were investigated. MiR124-MSCs group showed the ratio of NEUN(+) is (58.3% ± 2.3%), and TUJ-1(+) is (58.9% ± 1.8%) when compared with the control group (NC-group); miR21-5p-MSCs group showed the ratio of NEUN(+) is (49.1% ± 2.6%), and TUJ-1(+) is (64.0% ± 4.9%); MiRNA124+21-5p-MSCs group showed the ratio of NEUN(+) is (88.3% ± 1.5%), and TUJ-1(+) is (76.8% ± 1.9%). The group of miRNA124+21-5p-MSCs, miR124-MSCs and miR21-5p-MSCs showed significant differences with the group of NC-MSCs on the stained positive for NEUN and TUJ-1 expression. The group of miRNA124-MSCs have significant differences with the group of NC-MSCs on the stained positive cells for NEUN (***, *P*<0.0001) and TUJ-1 expression (***, *P*<0.0001). The group of miRNA21-5p-MSCs have significant differences with the group of NC-MSCs on the stained positive cells for NEUN (***, *P*<0.0001) and TUJ-1 expression (**, *P*=0.0045, <0.01). The miRNA124+21-5p-MSCs group have significant differences with the group of NC-MSCs on the stained positive cells for NEUN and TUJ-1 expression (***, *P*<0.0001). The proportion of stained positive cells for TUJ-1 (+) in miRNA124+21-5p-MSCs group was higher than that in MiRNA21-5p-MSCs group (*, *P*=0.0178, <0.05) and miRNA124-MSCs group (***, *P*<0.0001). The positive cell numbers of MiRNA21-5p-MSCs group and miRNA124-MSCs group have statistical difference compared with miRNA124+21-5p-MSCs group in NEUN (+) (***, *P*<0.0001) (Figure 4B,C).

## Discussion

In recent years, stem cell therapy using MSCs and gene therapy using miRNAs have received increasing attention in the treatment of SCI. However, only a very limited number (<2%) of transplanted MSCs successfully reach the injured tissues and can survive relatively a long time [39,40]. In order to improve the ability of cell migration, proliferation, and differentiation, we investigated the effect of miR124 and miR21-5p on MSCs migration, proliferation and neuronal differentiation. In this study, the results showed that miR124 can promote cell migration and miR21-5p can promote cell proliferation in MSCs. At the same time, both miR124 and miR21-5p can enhance the ability of MSCs to differentiate into neural cells.

Cell migration is an important determinant of the efficiency of MSC transplant therapy [41]. Previously, there are study reported that overexpression of miR124 suppresses the migration of several cell lineages, including glioma [42–44], bladder cancer cells [45,46], osteosarcoma cells [47,48], breast cancer cells [49,50], and lung cancer cells [51]. It is also reported that miR124 down-regulates Wnt/β-catenin signaling via targeting the frizzled-4 (FZD4) and low-density lipoprotein receptor-related protein 6 (LRP6) and thus suppresses the chemotactic migration of MSCs toward hepatocyte growth factor (HGF), a chemoattractant factor [52]. In the present study, via wound healing assay and transwell migration assay, our results indicated that overexpression of miR124 can promote the migration of MSCs. However, there are different results between our two experiments which might due to the different cell status and experimental conditions [52]. There are four time points (0, 6, 12, 24 h) in our study, while two time points (0, 12 h) were observed in the other experiment. In our experimental study, there was no significant difference when compared with the control at the 12 h time point, and it was not until the 24 h time point that the miR124-MSCs group showed significant different cell migration abilities [52]. Furthermore, overexpression of miR124 had no significant effect on the proliferation of MSCs, indicating that miR124 did not promote wound closure by increasing the cell population. It has been reported that miR124 may play an important role in neuroprotection and nerve function recovery in the early stages of spinal cord injury [53]. For example, overexpression of miR124 can ameliorate neuronal inflammation by modulating the anti-inflammatory M2 polarization in microglia after TBI and SCI [54,55]. Taken together, our data indicated that miR124 exhibit regulatory roles to promote MSCs migration *in vitro*, the target gene of miR124 and its functional roles *in vivo* still need to be further investigated experimentally.

It is reported that miR21-5p can enhance the migratory activity of glioma cells [56,57]. Furthermore, measured by transwell assay, miR21-5p contributes to MSCs migration by up-regulating matrix metalloproteinase-2 (MMP-2)/ matrix metallopeptidase 9 (MMP-9), potentially via the phosphatidylinositol-4,5-bisphosphate 3-kinase (PI3K)/Akt(Protein kinase B) pathway [58]. There are different results between our two experiments partly because of the different cells we used. We tested primarily MSCs extracted from rat bone marrow and the cell characteristics may be different between different cell sources [58]. Besides, it is also reported that miR-21 decreased cell migration and invasion of multiple myeloma cells by down-regulating sprouty homolog 2 (SPRY2) gene expression [59]. In this study, the results of wound healing assay and transwell migration assay both indicated that overexpression of miR21-5p showed no significant effect on MSCs migration.

The proliferation and survival of cells is another important factor to determine the efficiency of cell transplantation. There are reports showing that overexpression of miR124 can inhibit the proliferation of many types of cells, such as glioblastoma cells [44], cervical carcinoma cells [60], bladder cancer cells [45], liver cancer cells [61], osteosarcoma cells [48] and breast cancer cells [62]. While other reports suggested that miR124 promotes the proliferation of neural stem cells (NSCs) by targeting delta-like 4 (DLL4) [63]. As to MSCs, there are studies suggest that overexpression of miR124 reduces apoptosis of MSCs following oxygen and glucose deprivation [25]. It is also reported that overexpression of miR124 had no significant effect on the proliferation of MSCs within 12 h as evidenced by BrdU (Bromodeoxyuridine) incorporation assay which is consistent with our experiment measured by EdU staining [52].

Evidences have shown that miR21-5p plays a significant role in regulating cell proliferation and differentiation, whether miR21-5p can enhance or inhibit the proliferation of MSCs remains unknown, as scientists hold different perspectives on it. There are study indicated that miR21-5p had no effect on MSCs proliferation by using MTT and cell-cycle assay [58]. It is also reported that overexpression miR21-5p promote proliferation and inhibit apoptosis of MSCs, perhaps by down-regulating PTEN, via the PI3K/Akt pathway [29,64]. In addition, miR21-5p can enhance MSCs survival and can be transported to neurons through exosomes derived from MSCs and that it can target transient receptor potential melastatin 7 (TRPM7) to alleviate neuronal injury following intracerebral hemorrhage [65]. In our study, our data indicated that overexpression of miR21-5p can significantly promote MSCs proliferation. Furthermore, there is study suggesting that miR21-5p inhibitors can prevents cells from apoptosis in ischemic stroke by regulation of B-cell lymphoma-extra large (Bcl-xL), Caspase-3 and the 70 kilodalton heat shock proteins (HSP70) [66].

In previous studies, overexpression miR124 promoted differentiation and neurite outgrowth of NSCs with improved TUJ-1 expression and decreased GFAP expression [63]. In addition, miR124 was shown to increase expression of TUJ-1, MAP-2 and synaptophysin of MSCs after 6 days of *in vitro* culture [25]. Furthermore, miR124 can promote the neuronal differentiation of NPCs [27]. In this study, we found that miR124 can significantly increase the expression of TUJ-1 and NEUN in MSCs, indicating that miR124 promotes MSCs to differentiate into neurons *in vitro*.

MiR21-5p has been reported to promote angiogenesis and protect neurons from ischemic injury by different pathways including the FAS ligand (FASLG), Sprouty 2 and its target gene PTEN [67]. Overexpression miR21-5p in neural cells can increase the expression level of NEUN, TUJ-1 and GFAP [68]. It is reported that miR21-5p is transiently up-regulated in human adipose tissue-derived stromal cells (hASCs) during neural differentiation process and miR21-5p can promote the neural differentiation by binding to target sequences in the untranslated region of transforming growth factor beta receptor II (TGFBR2) [69]. MiR21-5p can also promote neural crest stem cells differentiate into Schwann cell and remyelination through down-regulating SOX protein expression by binding to the 3′-UTR of SOX2 mRNA [70]. Our results showed that miR21-5p can significantly increase the expression of TUJ-1 (**, *P*=0.0045, <0.01) and NEUN (***, *P*<0.0001) in MSCs, which indicated that miR21-5p promotes MSCs to differentiate into neurons *in vitro*. In addition, when overexpressing miR124 together with miR21-5p, the expression of TUJ-1 and NEUN in MSCs were significantly increased which indicated that miR124 and miR21-5p have a synergistic effect on neural differentiation of MSCs.

Collectively, our present experimental data indicated that miR124 can prominently promote cell migration and miR21-5p can significantly improve cell proliferation ability of MSCs. Both miR124 and miR21-5p can dramatically enhance the ability of MSCs to differentiate into neural-like cells. MiR124 and miR21-5p synergistically promote differentiation of MSCs into neurons. These results suggest that manipulation of the expression level of miR-124 and miR21-5p in transplanted MSCs may benefit cell migration and stem cell-based therapy prospectively. However, the identification of target genes of miR124 and miR21-5p, as well as their functional roles *in vivo* need to be investigated in the future experimentally.

## Abbreviations

BDNF: brain-derived neurotrophic factor
BME: β-mercaptoethanol
FZD4: frizzled-4
GDNF: glia cell-line derived neurotrophic factor
HGF: hepatocyte growth factor
IGF-1: insulin-like growth factor 1
LRP6: low-density lipoprotein receptor-related protein 6
MMP: matrix metallopeptidase
MSC: mesenchymal stem cell
NGF: neural growth factor
VEGF: vascular endothelial growth factor
